# Nasal Irrigation: An Imprecisely Defined Medical Procedure

**DOI:** 10.3390/ijerph14050516

**Published:** 2017-05-11

**Authors:** Nicola Principi, Susanna Esposito

**Affiliations:** 1 Pediatric Highly Intensive Care Unit, Fondazione IRCCS Ca’ Granda Ospedale Maggiore Policlinico, Università degli Studi di Milano, 20122 Milan, Italy; nicola.principi@unimi.it; 2 Pediatric Clinic, Università degli Studi di Perugia, 06123 Perugia, Italy

**Keywords:** allergic rhinitis, chronic rhinosinusitis, nasal irrigation, upper respiratory tract infection

## Abstract

Nasal irrigation (NI) is an old practice of upper respiratory tract care that likely originated in the Ayurvedic medical tradition. It is used alone or in association with other therapies in several conditions—including chronic rhinosinusitis and allergic rhinitis—and to treat and prevent upper respiratory tract infections, especially in children. However, despite it being largely prescribed in everyday clinical practice, NI is not included or is only briefly mentioned by experts in the guidelines for treatment of upper respiratory tract diseases. In this review, present knowledge about NI and its relevance in clinical practice is discussed to assist physicians in understanding the available evidence and the potential use of this medical intervention. Analysis of the literature showed that NI seems to be effective in the treatment of several acute and chronic sinonasal conditions. However, although in recent years several new studies have been performed, most of the studies that have evaluated NI have relevant methodologic problems. Only multicenter studies enrolling a great number of subjects can solve the problem of the real relevance of NI, and these studies are urgently needed. Methods for performing NI have to be standardized to determine which solutions, devices and durations of treatment are adequate to obtain favorable results. This seems particularly important for children that suffer a great number of sinonasal problems and might benefit significantly from an inexpensive and simple preventive and therapeutic measure such as NI.

## 1. Introduction

Nasal irrigation (NI) is an old practice of upper respiratory tract care that likely originated in the Ayurvedic medical tradition [1]. It was adopted by Western medicine in the late 19th century, and since then, it has continued to gain popularity worldwide [2]. It is used alone or in association with other therapies in several conditions, including chronic rhinosinusitis (CRS) and allergic rhinitis (AR) [3,4,5,6,7,8]. Moreover, particularly in children, it has been prescribed to treat and prevent upper respiratory tract infections (URTIs) [7]. In general, otolaryngologists and pediatricians consider NI very effective because its use has been found to be associated with a significant reduction in both the signs and symptoms of rhinosinusal diseases and the prescription of drugs commonly used in these conditions [9]. However, despite being prescribed in everyday clinical practice, NI is not included or is only briefly mentioned by experts in the guidelines for treatment of URTIs [10,11,12,13,14]. 

A great number of studies specifically planned to evaluate NI in clinical practice have been performed [7]. However, they frequently enrolled small numbers of patients with different ages and diseases and, in most of the cases, they had methodological problems leading to debatable results. Moreover, several aspects of NI, such as the composition of solutions, means of irrigation, mechanism of action, and safety and tolerability were not completely clarified. In this review, present knowledge about NI and its relevance in clinical practice will be discussed to assist physicians in understanding the available evidence and the potential use of this medical intervention. 

## 2. Mechanisms of Action of Nasal Irrigation (NI)

The exact mechanisms by which NI works are not known. However, most of the experts think that it is primarily a mechanical intervention leading to direct cleaning of the nasal mucosa, independent of the composition of the solution used for nasal washing [15]. The mucus lining the nasal cavity may be softened and dislodged. Moreover, inflammatory mediators—such as prostaglandins and leukotrienes—and antigens responsible for allergic reactions can be removed favoring resolution of URTIs and AR [16,17,18]. However, some data seem to indicate that the composition of the solution can influence some aspects of the NI action. Although the impact of the salt concentration on mucociliary clearance through a modification of ciliary beating frequency is not defined because data collected in vitro and in vivo have been contradictory, it has been demonstrated that the composition and activity of nasal secretions are related to the tonicity of the solution [19]. Administration of low-salt and isotonic solutions has been associated with an immediate, significant reduction in the microbial antigens and a related decline of microbial burden. In contrast, hypertonic solutions were found to be only marginally capable of influencing microbial antigen concentrations. Furthermore, lysozyme and lactoferrin concentrations were found to be increased by approximately 30% at 24 h after NI [20]. 

The activity of NI seems further increased by the addition to the solution containing ions different from Na^+^ and Cl^−^ because they can exert a relatively positive effect on epithelial cell integrity and function. Magnesium (Mg) promotes cell repair and limits inflammation by reducing the eicosanoid metabolism both at the level of the liberation of arachidonic acid and by direct inhibition of the 5-lipoxygenase enzyme [21]. Moreover, Mg inhibits exocytosis from permeabilized eosinophils [22] and, together with zinc, reduces apoptosis of respiratory cells [23]. Potassium exerts an anti-inflammatory action and, globally, all these ions seem to increase viability in respiratory cells more than isotonic saline [24,25]. Bicarbonate ions reduce mucus viscosity, although the relevance of the addition of pure bicarbonate to the saline solutions is debated [26]. The advantage of the reduced mucous viscosity might be counterbalanced by the increase in the pH of the solution, which might be a negative factor. In vitro studies have shown that acidic pH can reduce the ciliary beat frequency, whereas the opposite occurs when slightly alkaline solutions were used. However, in vivo, use of a solution with pH ranging from 6.2 to 8.4 did not affect mucociliary clearance [27]. Table 1 summarizes the mechanisms of action of NI.

## 3. Composition of Solutions Commonly Used for Nasal Irrigation (NI)

Isotonic saline (0.9%) and hypertonic saline (1.5% to 3%) are the most common commercial preparations used for NI. Both are acidic, with pH values varying from 4.5 to 7. Solutions with NaCl concentrations higher than 3% are not recommended, although the emergence of adverse events due to hypertonicity—such as sensations of pain, blockage, and rhinorrhea—have been demonstrated to be dose-dependent and occur only when the NaCl concentration is ≥5.4% [28]. Instead of traditional saline solutions, some physicians prefer Ringer’s lactate, which contains other minerals in addition to NaCl and has a pH from 6 to 7.5 [29]. To increase the mineral content, several commercial products containing seawater diluted with distilled water to obtain an isotonic or slightly hypertonic solution with neutral or slightly alkaline pH are on the market (Libenar^®^, Sterimar^®^, and Marimer^®^). Although diluted, these products include a greater amount of minerals compared to NaCl and Ringer’s solutions. An even greater content of ions is present in a product based on electrodialyzed seawater (Physiomer^®^) because this method of preparation maintains almost all the minerals of the original seawater. Solutions for NI can also be prepared at home according to the suggestions of several authors and institutions [19,30,31,32]. In general, boiled water mixed with table or canning salt is used. In some cases, baking soda is included. The final tonicity can vary from 0.9% to 3%, and the pH is acidic unless baking soda is added. 

## 4. Means of Nasal Irrigation (NI)

Several methods, including the one based on the old neti pot simply filled with lukewarm water, can be theoretically used to perform NI [33]. However, studies carried out on adults seem to indicate that among the various methods, the most effective are those that assure large volume irrigation. Moreover, although the optimal duration of the treatment has not been clarified, greater benefits result when positive pressure is used. The distribution of solution in nasal and sinus cavities is more exhaustive with positive pressure than with negative pressure (by sniffing), nebulization, or spray [34]. To maximize the efficacy, large-volume (no less than 100 mL) low-pressure irrigation is preferable to low-volume high-pressure irrigation [35]. Regarding devices, it has been established that, to allow the best irrigation of the whole nasal cavity and paranasal sinuses, compressible douching systems should be used. In adults, they should allow a minimum output pressure of 120 mbar, a good connection to the nostril, a possible insertion into the nasal vestibule, and an irrigation stream directed upwards (45°) [36].

In the community, the most often used device is a syringe. Unfortunately, a syringe has several limitations. First, it does not allow a good connection with the nostril. This reduces the efficacy of the NI because part of the solution may leak from the nostril before reaching the nasal cavity. Moreover, pressure can be quite different according to the force applied by the operator and, in some cases, may be too strong, which causes discomfort or too light, resulting in an ineffective application. Finally, when high volumes are needed and a relatively small syringe is used, it should be filled several times with the risk that the operator does not use the correct volume. 

Contrary to what has been precisely defined in adults, no evaluation of the most effective means of treating NI in children is available. This is a problem particularly for neonates, infants, and toddlers that cannot use compressive douching systems prepared for adults. In these patients, drops, sprays or disposable syringes are frequently used, although no study has precisely defined the best method, the right volume of solution, and the optimal duration of treatment to assure effective NI. Uncertainty surrounding NI in younger children is evidenced by the different types of NI used in children of a similar age suffering from the same disease. For example, in the study by Garavello et al. in which children with allergic rhinitis were evaluated, NI consisted of 2.5 mL of solution delivered to each nostril three times a day for six weeks with a syringe [37]. Marchisio et al. [38] and Chen et al. [39], treating children with a similar mean age and the same disease, used 20 mL 2 times a day with a syringe for 4 weeks, and 4–6 sprays twice a day for 12 weeks, respectively. All of these studies showed that NI was effective but did not document the best way to perform NI.

## 5. Tolerability and Safety of Nasal Irrigation (NI)

Adults generally have minimal side effects from NI. Transient adverse reactions, such as nasal irritation, nasal discomfort, otalgia, or pooling of saline in paranasal sinuses with subsequent drainage, have been described [31]. They are more common (10–20% of the cases) when very high volume devices are used. However, because they are mild in most of the cases, compliance is high [40]. Attention should be paid to the temperature of the solution because solutions that are either too cold or too hot can cause problems of tolerance. Similar conclusions can be drawn for children, although evaluation of compliance in these subjects is more difficult, particularly in the youngest patients. Tolerance is judged by parents and the evaluation is mainly derived from the opinion of the operator instead of the patient’s judgment. A detailed evaluation of tolerance and compliance of NI in pediatrics has been performed by Jeffe et al., who prescribed NI to 57 children aged 2–16 years after parents were instructed on how to rinse [41]. Each NI consisted of 100 mL of room temperature saline solution irrigated through each nostril for different periods according to parents’ judgment. Initially, 89% of children performed NI at least once a day, 7% performed NI one to four times per week, and 4% performed NI as needed. Parents were contacted between two and four months later and were asked to complete a questionnaire regarding the child’s experience. It was shown that 25% continued to use NI ≥ once daily, 5% one to four times per week, and 70% as needed. Compliance was high, independent of age, and was strictly dependent on initial parental assumption of tolerance. Only two children did not tolerate NI due to the emergence of adverse events. In six cases, NI was suspended because the child did not like the treatment, although significant adverse events did not occur. Among children who tolerated NI, 14% accepted the treatment after the first use, 73% in less than 7 days, and 11% in a period between 7 and 14 days. 

The problem of sterility of the solutions and devices has been debated. Solutions are at risk of contamination when large volumes of solution based on distilled water, bottled water, or boiled water are prepared at home, maintained in containers and used each time when NI is needed by withdrawing the required amount of liquid. Devices can be contaminated when they are continuously used without adequate cleaning [42]. Lee et al. reported that after one and two weeks of use, irrigation bottles used by adults undergoing endoscopic sinus surgery that were washed with hot soapy water after each use were found to be contaminated by a large spectrum of bacteria, including *Pseudomonas aeruginosa*, *Serratia marcescens*, *Proteus mirabilis*, and *Staphylococcus aureus* [43]. Similar findings were reported by other authors and, because in many cases contaminating bacteria were the same as those that could cause acute rhinosinusitis, it was suggested that the main source of device colonization was the sinonasal cavities [44,45]. The risk of contamination seems independent of the type of device [46]. Additionally, the use of a one-way valve irrigation bottle, theoretically capable of reducing the risk of reflux of contaminated solution in the device, was found to be practically ineffective [47]. In contrast, contamination seems to be influenced by the composition of the solution. It was shown that acidic, isotonic saline solutions were more frequently associated with bacterial contamination probably because some of the most common contaminants grow optimally in similar environmental conditions [48]. Finally, contamination was found more frequently with longer durations of NI use. With some exceptions, studies have reported that both bottles and bulb syringes were contaminated after one to two weeks of use in approximately 25% of the cases and in 45% after four weeks [48,49,50,51]. 

Although common and frequently based on potentially dangerous bacteria, contamination is considered a false problem by some experts [32]. They think that the nasal cavity is naturally full of bacteria and the addition of new pathogens is not clinically relevant. This conclusion seems supported by the evidence that contamination usually does not modify the disease outcome [48,49,50,51]. However, the evidence that replacement of old devices with new clean devices could improve symptom scores in adult patients with CRS [49] and, above all, the report of two primary amebic meningoencephalitis deaths associated with NI using contaminated tap water support the recommendation to pay attention to the problem of cleaning and sterilizing solutions and bottles for NI [52]. To limit contamination, several methods have been proposed and studied. Keen et al. compared several measures and found that rinsing devices with boiling water or disinfectant solutions or use of microwaves could reduce the degree of contamination in most, although not all, of the devices [49]. However, particularly when NI is performed at home, it seems mandatory to use only sterile, distilled, filtered water and rinse the device after each use using the same distilled or boiled water [52].

The risk of contamination has been studied practically only in adults. Because children mainly have sinonasal diseases due to pathogens different from those usually encountered in adults, no firm conclusions about the risk and type of contamination in pediatrics can be drawn. However, the same precautions recommended for adults are mandatory. Moreover, the use of sterile solutions delivered by spray or nebulization presently on the market and the use of sterile syringes discharged after each use could probably reduce the risk of contamination and related problems in children. When possible, nasal douches with warm sacs of premixed solution are recommended [53]. 

## 6. Clinical Efficacy

### 6.1. Acute Upper Respiratory Tract Infections (URTIs)

Acute URTIs, including the common cold and rhinosinusitis, are the most common diseases of children and are also extremely common in adults. Although mild diseases are observed in the greatest majority of patients, they significantly impact the health system and quality of life of patients and their families, especially when they are frequently recurrent, as usually occurs in the first years of life. To reduce signs and symptoms, antipyretic and decongestant drugs are frequently prescribed [54]. Moreover, even if they are mainly due to viruses, frequently they are treated with antibiotics for fear of superimposed bacterial infections. This leads to the abuse of antibiotics and all the problems related to the misuse of these drugs [55]. To limit the medical, social, and economic impacts of UTRIs and reduce drug consumption, NI is frequently prescribed by general practitioners, otolaryngologists, and pediatricians for both prevention and treatment. Several studies were planned to determine NI efficacy in URTI treatment. As previously reported, only a few had no risk of bias and could be used to draw reliable conclusions. This is clearly exemplified by a recent Cochrane review in which all the studies published until August 2014 comparing NI with at least one intervention or placebo for treatment of URTIs were initially included [56]. A total of 360 trials were retrieved, but among them, only five—two in adults and three in children—were selected for the final analysis because randomized controlled trials have risks of bias. Unfortunately, clinical characteristics of enrolled patients, prescriptions, and measures to evaluate treatment efficacy were not uniform and the results could not be pooled. Moreover, no information could be obtained on the relevance of the solution composition in the conditioning results. Consequently, firm conclusions could not be drawn. The authors concluded that NI might be a possible effective system for relieving the symptoms of acute URTIs, although further studies with greater numbers of participants and with more precise standardization of NI means and outcome measures are needed to confirm NI efficacy. Studies carried out in adults, enrolling a total of 205 subjects, reported no differences in nasal symptom scores between treatment and control groups [56,57]. A reduction in the time to resolution was evidenced by King et al., who found that the mean day of well-being for the group receiving isotonic saline solution was lower (7.67 days) than for the control group (10.48 days), although the difference did not reach statistical significance [56]. 

The results of pediatric studies were discordant. Bollag et al. enrolled 46 children aged two weeks to two years with various URTIs and assigned them to two intervention groups (saline nose drops; medicated nose drops) and a control group (no nose drops) [58]. Effectiveness was evaluated only two days later when it was found that a significant improvement in nasal signs and symptoms had occurred in all the groups without any advantage of saline drops. In contrast, positive results were reported by Slapak et al. who enrolled a total of 401 children aged 6–10 years with uncomplicated cold or influenza who were randomly assigned to two treatment groups, one with standard medication (i.e., antipyretics, nasal decongestants, and mucolytics) and the other with NI using electrodialyzed seawater solution [9]. The effect of treatment was evaluated by measuring the intensity of upper respiratory tract signs and symptoms after three weeks. The preventive effect of NI was evaluated considering the respiratory status and incidence of new URTIs together with the consumption of medication, complications, days off from school, and reported days of illness in the following nine weeks, during which NI was performed only three times per day compared to six times for the treatment period. It was shown that, in children with NI, both nasal secretion and nasal obstruction were significantly reduced (mean scores vs. control group, 1.79 vs. 2.10 and 1.25 vs. 1.58, respectively; *p* < 0.05 for both) at the end of the treatment period. Moreover, NI was found to exert a significant prophylactic effect because, in comparison to children without NI, children receiving NI for more than nine weeks had a lower need for using antipyretics (9% vs. 33%), nasal decongestants (5% vs. 47%), mucolytics (10% vs. 37%), and systemic antibiotics (6% vs. 21%; *p* < 0.05 for all). Moreover, in the same period, children in the saline group reported significantly fewer illness days (31% vs. 75%), school absences (17% vs. 35%), and complications (8% vs. 32%; *p* < 0.05 for all). A favorable result by Wang et al. was found for the use of normal saline in children with acute rhinosinusitis [59]. These authors enrolled 69 children aged 3–12 years. All received standard treatment. Thirty were also treated with NI with normal saline administered one to three times a day for three weeks. The efficacy of nasal treatment was evaluated considering the nasal peak expiratory flow rate (nPEFR) test, nasal smear examination, radiography (Water’s projection), and quality of life. After three weeks, compared to the control group, children with NI had an improved nPEFR test (*p* > 0.05) and there was a significant reduction of rhinorrhea, nasal congestion, throat itching, and cough and sleep quality improved. Similar advantages were also demonstrated in a subgroup of enrolled children; that is, patients with demonstrated atopy.

After publication of the Cochrane review, some other studies regarding NI and URTIs were published [49]. Among them, Regab et al. seemed to confirm the efficacy of NI in the treatment of URTIs. Sixty-two children with uncomplicated acute rhinosinusitis were enrolled in a prospective, randomized, placebo-controlled study [60]. The patients were enrolled into two groups, the first including children receiving amoxicillin and NI with normal saline solution and the second including patients treated with only NI. The same clinical response and the same middle meatus bacteriological and cytological changes were evidenced in both groups, suggesting that NI could be as effective as antibiotic treatment in acute mild rhinosinusitis of children. However, the previously drawn conclusions by the authors of the Cochrane review remain: the use of NI for URTI treatment is probably effective but we do not know how well it works, how it should be performed, what the best solution is for use and how long it has to be used for treatment and for prevention. 

### 6.2. Chronic Rhinosinusitis (CRS)

CRS is diagnosed mainly in adults and it is common in patients with underlying chronic disease involving the respiratory tract as cystic fibrosis or primary ciliary dyskinesia [61,62]. Most of the studies carried out to evaluate NI in this disease have been carried out in adult patients. To simplify treatment, usually based on antibiotics and corticosteroids, NI is frequently used. The clinical relevance of this therapeutic measure was first analyzed by Harvey et al. in a Cochrane review published in 2007, and it included 8 randomized controlled trials selected among the 64 studies initially retrieved [63]. As with the studies on treatment for URTIs, these case studies could not be globally pooled because of significant differences in patients, method of NI and outcome measures. Some studies included both pediatric patients and patients with allergic rhinitis. Saline solutions were compared with either no treatment, a placebo, as an adjunct to other treatments or against treatments. Hypertonic versus isotonic solutions were also compared. Conclusions were such that NI was capable of reducing symptom scores, although it was less effective than topical corticosteroids [63]. However, it can improve corticosteroid efficacy when used in combination. Finally, it was demonstrated that hypertonic and isotonic solutions had similar effects on patient symptoms and the quality of life, whereas hypertonic solutions were associated with significant improvement of radiological scores. More recently, a new attempt to evaluate the clinical relevance of NI in the treatment of CRS was made by Rudmik et al. [5]. These authors reviewed the studies carried out considering well defined primary clinical end-points that included only adult patients with disease diagnosed according to published diagnostic criteria. Randomized clinical trials of higher-quality studies were selected. However, lower-level studies were also considered if the topic contained insufficient evidence. Global quality was, however, poor. Eight trials, five performed in pre-surgical patients [30,46,64,65,66] and three [67,68,69] after endoscopic sinus surgery, were analyzed. Despite significant differences in the number of enrolled patients, the means and duration of NI and primary clinical end-points, a positive effect of the treatment, was evidenced in all the pre-surgical trials. Hypertonic and isotonic solutions were compared and found to be quite similar in relieving symptoms and improving endoscopy, mucociliary clearance, rhinomanometry, and olfactometry by Bachman et al., who treated a total of 40 patients with 200 mL of solution twice a day for one week [64]. However, differences in favor of normal saline were found by Hauptman and Ryan, who tested 80 patients with a single administration of 1 mL of buffered hypertonic or normal saline delivered through a metered-dose nasal spray bottle to the more symptomatic side of the nose [65]. Compared to basal conditions, both solutions improved the mucociliary clearance and symptoms of nasal stuffiness and obstruction. However, only buffered physiological solution could improve nasal airway patency. Finally, buffered hypertonic saline was more irritating.

Favorable results were also shown by two of the three post-operative studies, although with some limitations. Liang et al. reported that the addition of NI with isotonic saline solution to post-operative sinus debridement was effective in improving symptom scores only in patients with mild CRS [69]. Freeman et al. studied 23 patients comparing treatment with 2 mL of normal saline solution delivered via a mucosal atomization device for six weeks with no NI [68]. They found that the effect was transient because improved endoscopic appearance and mucociliary clearance were observed only after three weeks. In contrast, three months after intervention, only minimal and not significantly different variations were found with regard to crusting and edema, and there was no difference with adhesions, discharge and polyps. However, Pinto et al. did not report any advantage in the administration of both normal and hypertonic saline solutions in patients receiving NI with 30 mL four times a day [67]. 

A recent Cochrane review has confirmed that the quality of available publications regarding the use of NI in CRS is poor [70]. Only two studies were included. One [30] was already included in previous reviews [63] and only one new study was added [71], without significant improvement of our knowledge regarding all the problems related to NI use in CRS. In addition, data on different chronic diseases associated with CRS development are scant and do not permit to draw any conclusion.

### 6.3. Allergic Rhinitis (AR) 

AR is relatively common and it is a global health problem [72]. From a theoretical point of view, avoidance of allergens is the best measure to face AR, but it is often not feasible. To relieve acute signs and symptoms, anti-histamines and topical steroids are usually suggested [72]. However, all these drugs only help with the symptoms and do not provide long-term effects once treatment is suspended. Moreover, for some of them, long-term use can be followed by relevant adverse events. To overcome these problems at least partially, NI has been considered and several studies were planned to evaluate its real efficacy. A systematic review and meta-analysis of the studies published until 2010 [73] selected 10 trials [37,74,75,76,77,78,79,80,81,82], 7 of which were randomized and controlled studies. Three studies included children. However, once again, studies varied considerably with regard to many aspects involving patients, NI and evaluation of efficacy. Moreover, in some cases, a high risk of bias was evident. In general, NI was considered effective. Nasal symptom scores that were analyzed in eight of the studies improved in all the studied patients with variation from 3% to 70% in comparison to the baseline. Only pregnant women did not benefit from the treatment. Consumption of drugs was reduced, whereas mucociliary clearance times were improved. Comparison of isotonic versus hypertonic solutions led to discordant results. However, hypertonic solutions, with the exception of those based on special salts, such as Salsomaggiore or seawater gel [75] and the one used in the study by Garavello et al. [37], were associated with a mild worsening mucociliary clearance time and with a lower improvement of studied parameters in comparison to isotonic solution. However, the results of some studies carried out after the end of those included in the cited meta-analysis seemed to lead to different conclusions. The effectiveness of NI in reducing the signs and symptoms of AR was not always confirmed, whereas in some cases hypertonic solutions were found to be more effective. De Souza Campos Fernanades et al. compared 40 children with AR corticosteroid nasal spray administration to NI irrigation with isotonic saline solution [83]. Efficacy was measured through peak nasal inspiratory flow (PNIF) curves and a clinical score during the eight weeks of treatment and two weeks afterward. Corticosteroid administration was found to be significantly effective with increased PNIF percentages and lower clinical scores in most of the treated patients. In contrast, NI was only marginally effective: PNIF curves were not modified and the mean clinical symptom score was reduced by only 18%. Unsatisfactory results were also reported by Chen et al. [39]. These authors compared nasal corticosteroids with NI and with a combination of nasal steroids and NI. For NI, a physiological seawater solution was used. Treatment was given for 12 weeks and efficacy was measured through a score of nasal signs and symptoms and eosinophil quantification via a nasal smear. Combined treatment was the most effective, but NI alone was less effective than corticosteroids alone. Practically, in children with NI, nasal scores decreased only 10% and the eosinophil count was only marginally reduced. The use of a hypertonic solution was significantly more effective in the study carried out by Marchisio et al. [38], who confirmed what Garavello et al. had previously reported [37]. Marchisio et al. evaluated whether children with seasonal grass pollen-related AR could benefit from NI with normal saline solution or hypertonic solution (2.7% NaCl solution) by assessing the effects on nasal signs and symptoms, on middle ear effusion and on adenoidal hypertrophy [38]. Treatment lasted four weeks and, as in control patients, a group of children with similar clinical characteristics without NI was enrolled. Two hundred and twenty children (normal saline: 80; hypertonic saline: 80; no treatment: 60) completed the study. After four weeks, all the considered items were significantly reduced in the group receiving hypertonic saline (*p* < 0.0001), whereas in the group receiving normal saline, only rhinorrhea (*p* = 0.0002) and sneezing (*p* = 0.002) were significantly reduced. There was no significant change in any of the items in the control group. The duration of oral antihistamines was significantly lower in children receiving hypertonic saline than in those treated with normal saline or in controls [38]. 

In conclusion, regarding AR, the data did not allow firm conclusions to be drawn, although the high frequency of positive results seems to suggest a possible use of the NI in the treatment of AR.

## 7. Conclusions

Table 2 summarizes the main solutions, means of irrigation and indications of NI. Despite only one adequate study being carried out, and therefore being left unable to evaluate the preventive efficacy of NI [9], NI seems to be effective in the treatment of several acute and chronic sinonasal conditions. However, although in recent years several new studies have been performed, scientific evidence remains poor because most of the studies that have evaluated NI have relevant methodologic problems. This is demonstrated by the very small number of studies that were specifically performed to evaluate the impact of NI for the most common clinical conditions included in the Cochrane reviews. Only multicenter studies enrolling a great number of subjects, including those with chronic diseases as cystic fibrosis or primary ciliary dyskinesia, can determine the real relevance of NI, and these studies are urgently needed. Methods for performing NI have to be standardized to determine which solution, device, and duration of treatment are adequate to obtain favorable results. This seems particularly important for children that suffer from a great number of sinonasal problems and might benefit significantly from an inexpensive and simple preventive and therapeutic measure such as NI. 

## Figures and Tables

**Table 1:** Mechanisms of action of nasal irrigation (NI).

Mechanism	Action
Mechanical intervention	Mucus lining dislodgment
	Removal of inflammatory mediators
Impact on mucociliary clearance	Reduction in microbial antigens level
	Decline of microbial burden
Positive effect on epithelial cell integrity and function in the presence of additional ions	Magnesium promotes cell repair, limits inflammation, limits exocytosis, and reduces apoptosis of respiratory cells
	Zinc reduces apoptosis of respiratory cells
	Potassium exerts anti-inflammatory action
	Bicarbonate reduces mucus viscosity

**Table 2:** Main solutions, means of irrigation, and indication of nasal irrigation (NI).

Mechanism	Action
Most common composition	Isotonic saline (0.9%) or hypertonic saline (1.5–3%)
	pH varying from 4.5 to 7
Optimal means of irrigation	Large volume
	Positive pressure
	Compressible douching system
Indication	Acute upper respiratory tract infections
	Chronic rhinosinusitis
	Allergic rhinitis
